# Immunomonitoring of Human Breast Milk Cells During HCMV-Reactivation

**DOI:** 10.3389/fimmu.2021.723010

**Published:** 2021-09-09

**Authors:** Katrin Lazar, Thorsten Kussmann, Graham Pawelec, Simone Pöschel, Rangmar Goelz, Klaus Hamprecht, Kilian Wistuba-Hamprecht

**Affiliations:** ^1^Institute for Medical Virology and Epidemiology of Viral Diseases, University Hospital Tübingen, Tübingen, Germany; ^2^Department of Immunology, Interfaculty Institute for Cell Biology, University of Tübingen, Tübingen, Germany; ^3^Cancer Solutions Program, Health Sciences North Research Institute, Sudbury, ON, Canada; ^4^Flow Cytometry Core Facility, Medical Faculty, University of Tübingen, Tübingen, Germany; ^5^Department of Neonatology, University Children’s Hospital Tübingen, Tübingen, Germany; ^6^Section of Dermatooncology, Department of Dermatology, University Medical Center, Tübingen, Germany; ^7^Section for Clinical Bioinformatics, Internal Medicine I, University Medical Center, Tübingen, Germany

**Keywords:** human cytomegalovirus (HCMV), lactation, breastfeeding, T cells, B cells, phenotyping, (imaging) flow cytometry

## Abstract

**Background:**

Breast milk leukocytes may play a role in protecting the infant from pathogens. The dynamics and the role of lymphocytes in human cytomegalovirus (HCMV)-seropositive mothers shedding HCMV into breast milk during the first months postpartum (p.p.) are mostly unclear.

**Methods:**

Breast milk cells were analyzed by Pappenheim panoptic and alpha-naphthyl acetate esterase staining as well as by imaging and polychromatic flow cytometry to simultaneously establish their morphological and phenotypic properties. The latter were characterized in HCMV-seropositive and seronegative mothers´ breast milk cells at different time points p.p.

**Results:**

Panoptic staining of breast milk cells revealed the presence of monocytes/macrophages, granulocytes and lymphocytes. Imaging flow cytometry data combining phenotypic and morphological analysis identified NKT-like cells, NK cells, epithelial cells, T cells and monocytes/macrophages. HCMV-seropositive but not -seronegative mothers had significantly higher T cell frequencies in mature milk.

**Conclusions:**

The presence of lymphocyte subsets in breast milk may be more influenced by the HCMV-seropositivity of the mother than previously recognized.

## Introduction

Breast milk is very important for newborns as nutrition but also to support their immature immune system (1, 2). Relatively high percentages of the thus far identified 976 proteins in milk are involved in immune functions (3, 4). From immunoglobulins, oligosaccharides, proteases to cytokines, breast milk is well equipped to defend against pathogens even in the infant’s gut (1, 5). A potentially important mode of immune support may also be the cellular components present in breast milk. Breast milk cells consist mainly of myo-epithelial cells (6), alveolar epithelial cells defined as lactocytes, stem cells, progenitor cells, monocytes, macrophages, granulocytes, T cells, B cells, NK and NKT-like cells. The percentage of leukocytes is highest in colostrum with a very high inter-individual variation of 13-70% of total breast milk cells (7). In transitional (days 8-30 p.p.) and mature milk (> 30 days p.p.), the leukocyte frequencies decrease to 0-2% of total milk cells in healthy mothers (8). However, the percentage of leukocytes may change dramatically if the mother is infected with a pathogen, and can increase rapidly up to >90% of total breast milk cells (7). With respect to the highly variable reaction of the cellular (8) and humoral (9) immune system to pathogens, acquired by the mother, here we focus on the impact of human cytomegalovirus (HCMV) reactivation during lactation.

HCMV is a β-herpesvirus (HHV5) with an important pathological role in the setting of solid organ and hematopoietic stem cell transplantation, but infection is mostly asymptomatic in individuals who are not immunosuppressed (10). HCMV natural latency is established in bone marrow precursor cells (CD34+) but reactivation occurs in differentiated macrophages and dendritic cells (11). In contrast, HCMV reactivates in immunocompetent healthy breastfeeding women and is present in breast milk, but the mechanism for compartmentalized unimodal HCMV reactivation and shedding into breast milk is poorly understood. Earlier studies reported that viral reactivation during lactation occurs in nearly all HCMV-seropositive women (12). HCMV-infected CD14+ monocytes/macrophages isolated from breast milk may be responsible for viral transmission (13). Recent preliminary findings showed that certain cytokines such as CXCL10 may be involved in the dynamics of the early HCMV reactivation process during lactation, *via* induction of a proinflammatory cytokine shift (14). Interestingly, HCMV reactivation occurs locally in the mammary gland in the majority of seropositive immunocompetent breastfeeding mothers without detection of viral DNA in blood or urine (15). Breast milk viral loads can vary greatly with up to 2.6x10^6^ copies/ml (9).

Preterm infants at risk, which include preterm infants with gestational age <32 weeks and under 1500 g, easily become infected with HCMV and may experience devastating consequences including colitis or sepsis-like symptoms (16). Recently, a new short term heat inactivation method based on the generation of a milk film was developed to prevent mother-to preterm infant transmission, as shown by *in vitro* experiments and a bicentric controlled prospective clinical study (17).

The cellular immune response to HCMV in peripheral blood is mainly under the control of CD8+ T cells and NK cells (18–20). Our focus in the present study was to standardize analysis methods and to apply them for the determination of the frequencies of these immune cell populations in HCMV-seropositive and –negative mothers’ breast milk. To this end, we isolated breast milk cells at different times after birth and analyzed them simultaneously by panoptically-stained cytospin preparations, imaging flow cytometry and polychromatic flow cytometry.

## Materials and Methods

### Study Design

Breastfeeding, healthy HCMV-seropositive (n=8) and –seronegative mothers (n=7) of either preterm or full-term infants were invited to join the study at the Neonatology Department of the University Hospital Tübingen between May and September 2016. The demographics of the 15 mothers participating in the study are shown in **Table 1**. Breast milk samples were taken at different time points: T1 was chosen ranging from the end of colostrum production on day 6 to the end of transient milk on day 30 p.p., and T2 after 30 days p.p. (mature milk). Mothers mostly donated breast milk at either T1 or T2. Additionally, sequential samples of three mothers were collected with one sample either at T1 or T2 (**Table 1**). All mothers had given their written informed consent and the study was approved by the Clinical Ethics Committee at the University Hospital Tübingen (804/2015BO2).

**Table 1:** Demographics of the breastfeeding mothers.

Mother	Age	GA	BW	HCMV	BM sampling	Viral load[copies/ml] or nPCR*	Cohorts
	[years]	[week+days]	[g]	IgG	[days pp]	
1	36	37 + 4/7	2680	neg	11	neg*	T1
2	33	38 + 4/7	3230	neg	12	neg*	T1
3	40	35 + 6/7	1195	neg	16	neg*	T1
4	31	30	1140 + 1240*	neg	44	neg*	T2
5	31	27 + 3/7	923 + 945*	neg	57	neg*	T2
6	31	27 + 3/7	1150	neg	78	neg*	T2
7	34	30	1630	neg	**12**, 41, 47, **54**, 67	neg*; neg*; neg*; neg*; neg*	**T1,T2,** longitudinal
8	29	35 + 5/7	2250	pos	6	neg*	T1
9	30	34 + 4/7	2650	pos	12	545	T1
10	30	33 + 2/7	1985	pos	17	2,050	T1
11	39	40	4110	pos	20	84,400	T1
12	44	29 + 1/7	1145	pos	39	10,200,000	T2
13	35	24 + 2/7	590 + 595*	pos	**108**,	1,800;	T2
					(57, 122, 131)	(1,170,000; 36,800; 3,260)	
14	33	34 + 5/7	2515	pos	13, **22**, **42,** 68	0; **0**; **0**; 0	**T1,T2,** longitudinal
15	29	27	900+710+975 twins	pos	**20**, 48, 55, **62**	**0**; 21,500; 10,300**; 2,680**	**T1,T2,** longitudinal

GA, gestational age; BW, birth weight; BM, breast milk.

Bolded BM sampling time points for cellular analysis correspond to viral load and Cohorts.

*Multiple births.

### HCMV-Serostatus, Breast Milk Cell Isolation and HCMV Monitoring in Breast Milk

HCMV-serostatus of all breastfeeding mothers was determined using maternal serum at birth for an electrochemiluminescence immunoassay ECLIA (ECLIA-Elecsys CMV-panel, cobas6000 module e601; Roche, Basel, Switzerland). Eight mothers were HCMV-seropositive and seven were HCMV-seronegative.

Pumped breast milk not older than 13 hours was collected and the cells were isolated. In brief, the samples were centrifuged at 400g for 10 min at 4°C, the top fat layer was discarded, and the middle whey layer was centrifuged again at 1780g for 10 min followed by a sterile-filtration step with a 0.22µm pore sized filter to gain milk whey (21). The milk cell pellet was washed twice with PBS, viable cells counted by trypan blue dye exclusion and used for cytospin panoptic staining after Pappenheim, or alpha-naphthyl acetate esterase staining, or for imaging and polychromatic flow cytometry.

Breast milk viral loads were monitored in milk whey using nested PCR (nPCR) with a limit of detection (LOD) of 200 copies/ml as described earlier (22), as well as by quantitative real-time PCR of the UL83 gene using Light Cycler 2.0 (Roche, Basel, Switzerland, LOD 600 copies/ml) and the CMV R-gene^®^ Kit (Biomérieux, Marcy-l’Étoile, France). In cases of HCMV-seronegative mothers, nPCR with lower LOD was performed from breast milk, while in seropositive mothers quantitative real-time PCR data are given.

### Cytospin Preparations

For the cytospin preparations, 1.5x10^4^ of isolated breast milk cells was centrifuged (28g) using Cytospin3 (Shandon, USA) onto a microscopic slide and stained either for Pappenheim (May-Gruenwald and Giemsa solution from Merck KGaA, Darmstadt, Germany) or for α-naphthyl acetate esterase (α-naphthyl acetate esterase kit from Sigma-Aldrich, St. Louis, USA).

### Flow Cytometry and Imaging Flow Cytometry

Multispectral Imaging Flow Cytometry combines the features of fluorescence microscopy and conventional flow cytometry and enables an objective discrimination of cells based on their shape and phenotype. For imaging flow cytometry, the following cell surface markers were used for characterization of breast milk cells: CD45-BV510 (HI30, BD Biosciences, USA), CD3-APC (UCHT1, Biolegend, USA), CD19-BV605 (SJ25C1, BD Biosciences, USA), CD14-PE-Cy7 (63D3, Biolegend, USA), CD16-Pacific blue (3G8, Biolegend, USA), CD66b-FITC (G10F5, Biolegend), CD326-PE (9C4, Biolegend, USA), CD56-APC-H7 and HLADR-PerCPCy5.5.

In an initial 20 minutes incubation, cells were stained with ethidium monoazide bromide (EMA) from Biotium, (Haywar, California, USA) for evaluation of cell viability, together with polyclonal immunoglobulins (GAMUNEX, Grifols, Barcelona, Spain) to block non-specific Fcγ-receptor binding. The antibody mix for subsequent staining included the markers mentioned above. At least 10.000 cells were acquired for each sample with 60x magnification using the ImageStreamx mkII with INSPIRE instrument controller software in the Imaging Flow Cytometry Core Facility of the University Hospital Tübingen and analyzed with IDEAS^®^ (Image Data Exploration and Analysis Software, both from Luminex Corporation, Austin, Texas, USA, version 6.2).

Polychromatic flow cytometry was conducted using isolated breast milk cells and a biological control (frozen PBMC of the same single reference donor) on each measuring day. After the same EMA/GAMUNEX treatment as described above, the antibody mix for subsequent staining consisted of CD45-BV510 (HI30, BD Biosciences, USA), CD3-APC (UCHT1, Biolegend, USA), CD19-BV605 (SJ25C1, BD Biosciences), CD56-APC/FireTM 750 (5.1H11, Biolegend, USA) and HLADR-PerCPCy5.5 (G46-6, BD Biosciences). Cells were then analyzed on an LSR II flow cytometer (BD, USA). Compensation controls were measured for each experiment for correcting spectral overlap. Analysis of the data was accomplished with FlowJo (BD, version 10.6.1). The gating strategy is shown in **Supplementary Figure 1**.

### Statistical Analysis

The two time points T1 and T2 of breast milk lymphocyte subpopulation analysis from mothers of the same HCMV-serostatus were analyzed with the Mann-Whitney U-test. Time ranges from mothers of different HCMV-serostatus were also compared using Mann-Whitney U testing. Significance level was set at p<0.05.

## Results

### Cell Morphology and Phenotype in Human Breast Milk

**Figure 1:**
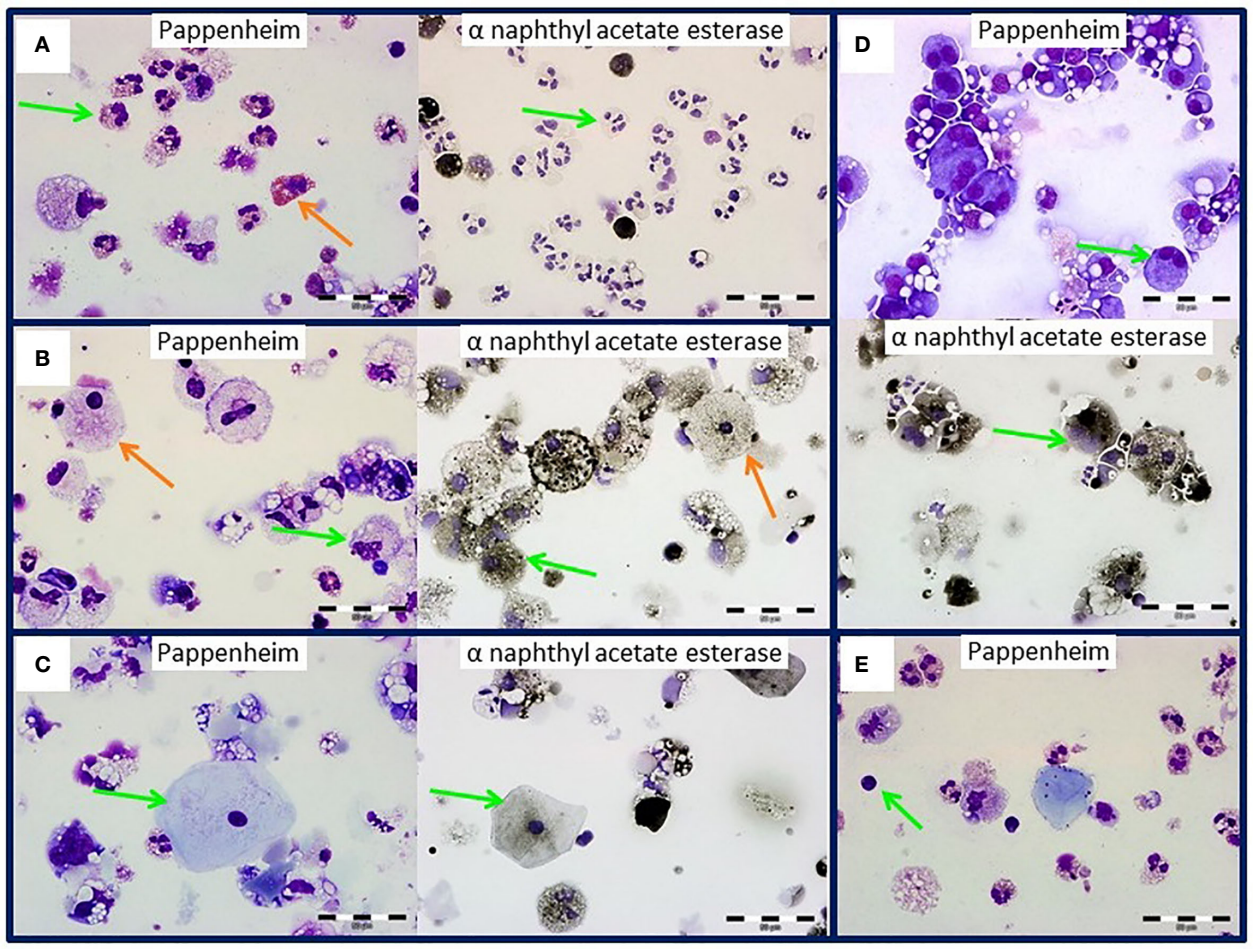
Breast milk cells of an HCMV-seropositive mother. Green arrows indicate **(A)** granulocytes, **(B)** monocytes, **(C)** epithelial cells, **(D)** monocytes with two nuclei and **(E)** lymphocytes by panoptic Pappenheim (left) and α-naphthyl acetate esterase (right) staining. Orange arrows show eosinophils **(A)** or lactocytes **(B)**. Images at 600x magnification.

Breast milk cells were immobilized on microscope slides and either stained panoptically or for α-naphthyl acetate esterase (**Figure 1**, left and right images respectively). Neutrophilic granulocytes (**Figure 1A**, green arrows), monocytes/macrophages (**Figure 1B**, green arrows), epithelial cells (**Figure 1C**, green arrows) and lymphocytes (**Figure 1E**, green arrow) were identified. The morphological characteristics suggest a lactocyte in **Figure 1B** (orange arrows). Almost all cells showed high granularity and multiple fat inclusion bodies. As shown in **Figure 1D** we could also identify cells with two nuclei.

**Figure 2:**
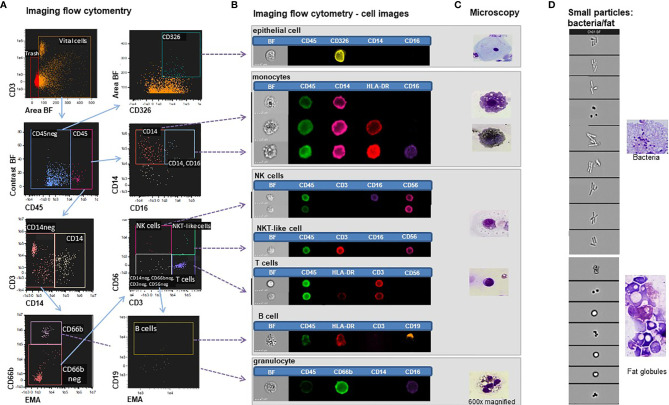
Imaging flow cytometry of breast milk cells. **(A)** Gating strategy in IDEAS^®^ of breast milk cells stained with EMA, CD45-BV510, CD3-APC, CD56-APC-H7, CD14-PE-Cy7, CD16-Pacific blue, CD66b-FITC, CD326-PE and HLADR-PerCPCy5.5. **(B)** Bright field (BF) and fluorescent images of epithelial cells, monocytes, NK cells, NKT-like cells, and T cells, B -cells and granulocytes. **(C)** Microscopy images (600x magnification) of the corresponding panoptic staining of cytospin preparations. **(D)** Small particles excluded as trash in the imaging flow cytometry experiments (left panel) showing morphologic characteristics of bacteria and fat globules compared to panoptically stained images (right panel).

Breast milk cells showed diversity in morphology compared to commonly known peripheral blood cells, which do not display for example fat inclusion bodies. In order to link the cellular morphology of breast milk immune cells, as identified under the microscope, with characteristic leukocyte surface marker expression profiles, an imaging flow cytometry analysis was performed. Serial gating on the respective markers identified the immune cell populations of interest that were then further studied for their morphology using the acquired cell images (**Figure 2**). **Figure 2A** displays the applied gating strategy starting with viable, single cells in focus. Areas of bright-field versus intensity of CD3 were used to identify immune cells and exclude small particles. Next, CD14+ monocytes were identified out of all CD45+ leukocytes which were further divided into classical monocytes (CD45+ CD14+ HLA-DR+) and intermediate monocytes (CD45+ CD14+ HLA-DRbright CD16dim). Putative mMDSCs were characterized as CD45+ CD14+, HLA-DR- CD16- (**Figure 2B**).

The granulocyte populations were identified as CD45+ CD14- CD66b+. By plotting CD56 against CD3, we further divided the CD66b-negative population into NK cells (CD45+ CD3- CD56+), NKT-like cells (CD45+ CD3+ CD56+) and T cells (CD45+ CD56- CD3+). B cells were identified as CD45+ CD3- CD56- CD19+.

We also identified T cells (CD45+ CD3+ CD56-), activated T cells (CD45+ CD3+ HLA-DRdim), NKT-like cells (CD45+ CD3+ CD56+) and putative resting (CD45+ CD3- CD56+ CD16-) as well as activated NK cells (CD45+ CD3- CD56dim CD16+). Activated B cells (CD45+ CD3- CD19+ HLA-DR+) were also identified. Furthermore, granulocytes could be divided in CD16high (presumably mature neutrophils) and CD16low (e.g. eosinophils) subsets. We also found epithelial cells (CD45- EpCAM+[CD326]) in breast milk with similar morphology as seen in the cytospins. Due to size and morphology, we did not further investigate these cells in the following flow cytometry experiments. Comparative analyses of cell morphology in the bright field and the fluorescence images with the microscopy images at the single cell level identified different myeloid and lymphoid populations as well as granulocytes (**Figures 2B, C**). Interestingly, also the very small particles that are usually excluded as “trash” in flow cytometry experiments was found to contain bacteria and fat globules in addition to cellular debris, as also seen in microscopic evaluation of cytospin preparation (**Figure 2D**). Thus, these analyses proof, that breast milk derived immune cells can be studied using established lineage markers, despite divergent morphology compared to commonly known peripheral blood cells.

### HCMV Reactivation in Breast Milk

No HCMV DNA was detected by nPCR of milk whey from the 7 breastfeeding HCMV-seronegative mothers in this study at any of the time points of milk sampling (**Table 1**). In contrast, viral DNA was detected in cell- and fat-free milk whey in 6 of 8 HCMV-seropositive breastfeeding mothers (**Table 1**). However, in one instance (case 8) only late colostrum from day 6 p.p. corresponding to T1 could be analyzed prior to the assumed onset of unimodal viral reactivation. Breast milk of mother 9, collected on day 12 p.p. showed low viral load (VL) close to LOD, but outside the linear amplification curve. Mother 14 was the only seropositive breastfeeding mother in whom no viral DNA could be detected in milk whey over the whole observation period including T1 and T2. Five of the 8 seropositive mothers shed HCMV into breast milk with VL ranging from 2x10^3^ copies/ml to 10^7^ copies/ml of milk whey.

### HCMV-Seropositivity Associates With Breast Milk Lymphoid Cell Frequencies

**Figure 3:**
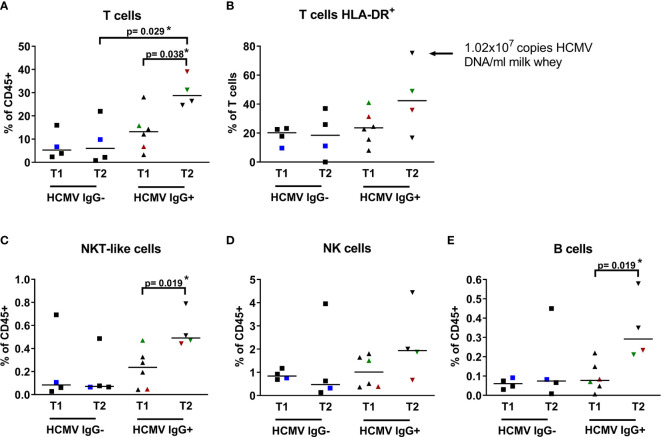
Breast milk lymphocyte subpopulations of 15 HCMV seropositive and seronegative mothers. Frequencies within CD45+ leukocytes of **(A)** T cells (CD45+ CD3+ CD56-), **(B)** activated T cells (CD45+ CD3+ HLA-DRdim), **(C)** NKT-like cells (CD45+ CD3+ CD56+), **(D)** NK cells (CD45+CD3-CD56dim), **(E)** B cells (CD45+ CD3- CD19+) of HCMV-IgG- and IgG+ mothers at two time points (T1: 6-22, T2 39-108 days p.p.). Mothers with consecutive samples are color coded (blue: mother 7, red: mother 14 and green: mother 15). Statistical analysis was performed by Mann-Whitney U-test. The arrow in **(B)** highlights very high HLA-DR expression on CD3 T cells of one mother.

After investigating key markers for prominent cell subsets in breast milk, we focused on the lymphoid cell subsets which are mainly involved in the HCMV immune response. Therefore, we made use of our established breast milk immune cell phenotyping approach as described above and quantified T cell, NK cell and B cell populations in 15 mothers (8 HCMV+ and 7 HCMV-) at two time points during lactation. To study effects of HCMV on these breast milk cells, the participating mothers were divided into groups by HCMV-serostatus and two time ranges (T1<30 days p.p., T2>30 days p.p.). Each time range (T1, T2) included at least four individual milk specimens (**Figure 3**). Mothers 7, 14, and 15 with longitudinal sample collection had several time points at T2. For **Figure 3** the data points at T2 of the HCMV-seropositive mothers (14 and 15) were selected after the (supposed) viral peak load in the descending arm of the unimodal reactivation pattern (23), which might coincide with increased T-cell activity leading to the end of viral reactivation. For the HCMV-seronegative mother, the data for all samples in T2 were taken together and the average is shown in **Figure 3**.

Interestingly, there was no difference in the monitored immune cell composition of transitional breast milk samples (T1) between HCMV-seropositive and -negative mothers. In contrary to this finding, mature breast milk samples (T2) revealed higher T cell frequencies in HCMV-seropositive compared to seronegative mothers (p=0.029, **Figure 3A**). Also, the frequency was significantly higher in seropositives but not in seronegatives comparing T1 (IQR 6.63) and T2 (IQR 7.07; p=0.038; **Figure 3A**). Additionally, activated T cells expressing HLA-DR tended to be more frequent in seropositive mothers at T2 (IQR 23.73) than T1 (IQR 14.36, p=0.25), and the mean frequency also tended to be higher than in seronegative mothers at T2 (IQR 22.42, p=0.20, **Figure 3B**). Unexpectedly, one of the HCMV-IgG+ mothers exhibited unusually high frequencies of this activation marker (on 75% of all T cells, **Figure 3B**, arrow). Interestingly, this mother showed an extraordinary high VL of 1.02x10^7^ copies/ml. Frequencies of certain other HCMV-associated lymphocyte populations such as NKT-like (p=0.11), NK cells (p=0.2) and B cells (p=0.2) also tended to be present at slightly higher frequencies at T2 in seropositives than seronegatives (**Figures 3C–E**). When comparing T1 to T2 only in seropositive mothers, a significant increase of frequencies was apparent for NKT-like cells and B cells (p=0.019, **Figures 3C, E**). In **Supplemental Table 1** all frequency data of lymphocyte subpopulations of all 15 mothers including IQRs are summarized.

Additionally, comparing lymphocyte frequencies between the HCMV IgG seronegative and HCMV seropositive subcohorts using all available time points, showed significantly higher frequency of CD45+CD3+ T cells (p=0.021) in the seropositive subcohort (**Supplemental Figure 2**).

In the small subcohort of seropositive mothers no correlation between VL and frequencies of lymphocyte subpopulations were found.

### An Example of Longitudinal Investigation of Breast Milk-Derived Lymphoid Cell Phenotypes and Viral Load

**Figure 4:**
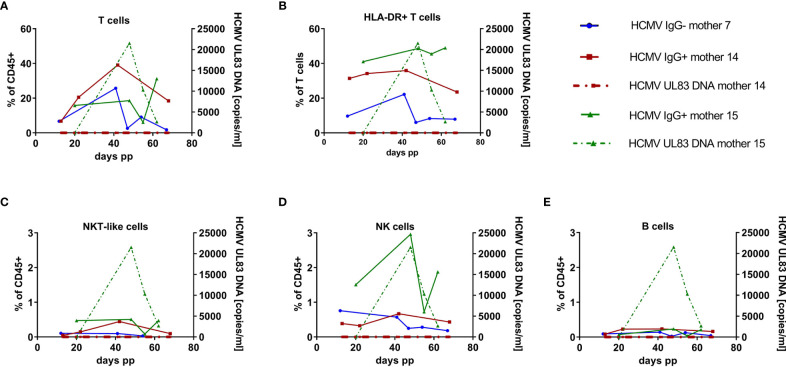
Longitudinal case studies of 3 mothers. **(A)** Breast milk T cell (CD45+ CD3+ CD56-), **(B)** HLA-DR-positive T cell (CD45+ CD3+ HLA-DRdim), **(C)** NKT-like cell (CD45+ CD3+ CD56+), **(D)** NK cell (CD45+CD3-CD56dim) and **(E)** B cell (CD45+ CD3- CD19+) frequencies of one HCMV-seronegative (mother 7, blue), and two HCMV-seropositive mothers (mother 15, green, with HCMV-DNA in breast milk and mother 14, red, *without HCMV reactivation in breast milk). Dotted lines present HCMV UL83 DNA viral load in milk whey.

Frequencies of the above described lymphocyte subpopulations of three mothers (HCMV-seronegative mother 7 and HCMV-seropositive mothers 14 and 15) were analyzed longitudinally at 4/5 time points after birth, respectively, and are displayed together with the viral load in **Figure 4**. Unexpectedly, mother 14 did not reactivate HCMV during the observed times in milk whey, whereas mother 15 showed the expected unimodal course of viral shedding (9, 23) with peak viral load at 48 days p.p. with 2.15x10^4^ copies HCMV DNA/ml of milk whey. All 3 mothers showed high interindividual variations over time in all observed lymphocyte subsets. However, and anecdotal due to the low number of participants, mother 15 (HCMV reactivation) had the highest proportion of activated T cells over time, followed by mother 14 (no HCMV reactivation, but seropositive) and mother 7 (HCMV-seronegative), with the lowest frequencies of these cells.

Seropositive mother 14 without any HCMV reactivation during the observation period had the highest T cell frequencies with a peak at 42 days p.p. (**Figure 4A**). Activated HLA-DR-expressing T cells were highest in mother 15 and lowest in the HCMV-seronegative mother 7 (**Figure 4B**). Seropositive mother 15 also had elevated frequencies and peak levels of NK cells (**Figure 4D**). NK cells showed an increase from about 1.5 to 3% from day 20 to day 48 p.p., the time point with peak viral load. NKT-like cells and B cells were found only at very low frequencies (<0.5%) of all leukocytes in breast milk, without relation to HCMV-serostatus and viral load (**Figures 4C, E**). None of the analyzed lymphocyte subpopulations correlated with HCMV VL based on the individual longitudinal course of mother 15.

## Discussion

In this pilot study, we established a high-throughput immunomonitoring approach to study the numerically rare human breast milk leucocyte populations that are thought to be involved in the protection of the newborn against microbes (8). First, we established a gentle breast milk cell isolation protocol, followed by morphologic studies using Pappenheim panoptic and alpha-naphthyl acetate esterase staining. This was technically challenging in many ways, especially because some breast milk leucocytes were difficult to identify in cytospin preparations due the vast majority of total cells not being leucocytes. Rather, they were epithelial cells (6) (note that the epithelial cell shown in **Figure 1** may be categorized as a skin-derived cell, as it had no inclusion bodies reflecting the influence of breast milk pumping), either ductal cells or lactocytes from the alveoli producing milk, and also myoepithelial cells. Both lactocytes and monocytes/macrophages contained multiple inclusion bodies and were sometimes difficult to distinguish from one another. Even the α-naphthyl acetate esterase staining, which should stain monocytes and to a lesser degree lymphocytes (24, 25), did not help in distinguishing monocytes from lactocytes, because the latter also seemed to stain slightly for α-naphthyl acetate esterase. Granulocytes (eosinophils and neutrophils) as well as lymphocytes were easy to distinguish, although no further division of the lymphocytes into T or NK cells was possible. Cells with two nuclei could also be observed, which we believe to be polynuclear macrophages or in some cases also ductal epithelial cells still connected with tight junctions (26, 27).

For further subset analyses of the morphologically diverse breast milk leucocytes, we applied imaging flow cytometry that allows bright field imaging of the cells and simultaneously their characterization by immunostaining (28). Using this approach, CD14+ monocytes/macrophages were clearly distinguishable from CD326+ epithelial cells, CD3+ T cells, CD56+ NK cells or CD66b+ granulocytes. We could also identify CD19+ B cells, albeit at very low frequencies. In addition to the above discussed advantages of the imaging flow cytometry technology for the investigation of defined breast milk leukocyte subsets, we also found morphological evidence for bacteria in the population that contains particles of relatively small size, which is usually excluded as ‘trash’. This result is supported by the finding of many different bacteria in breast milk which might also derive from the breast skin surface (29) with typical cocci- and rod-shapes. Taken together, our comparative panoptic and imaging flow cytometry analyses demonstrate the feasibility of high throughput analyses of human breast milk samples *via* polychromatic flow cytometry.

In contrast to the settings of solid organ transplantation and stem cell transplantation, HCMV reactivation during lactation is characterized by local self-limited virus shedding into breast milk after birth and during the following 2-4 months. HCMV DNA cannot be detected during this time period in blood, urine or saliva. In the present study, HCMV reactivation in seropositive breastfeeding mothers was defined by detection of viral DNA in the whey fraction of breast milk using milk samples mostly from transient and mature milk. We were able to examine only one milk sample derived from day 6 p.p. (mother 8), which represents the end of colostrum production. At this early time point, it is known that only a minority of breastfeeding seropositive mothers reactivates the virus, as recently shown by a detailed statistical analysis (23). None of the 7 seronegative mothers tested here showed any evidence of viral shedding. In contrast, in seropositive mothers we detected viral DNA at different time points in 6 of 8 breastfeeding mothers, with a viral load ranging from close to LOD to 10^7^ copies HCMV DNA/ml of milk whey. Only one seropositive mother failed to yield any evidence for viral reactivation during the observation period. This is not unusual, because it has been reported that not exactly 100% of seropositive breastfeeding mothers shed the virus into milk (9, 12, 30).

The mother-to infant-transmission rate for preterm and term infants ranges between 30 and 40%. Infected preterm infants may be severely harmed by postnatal HCMV infection (15, 31). To prevent postnatal HCMV transmission, a new method for short term-heat inactivation of HCMV using milk films was developed (23). Therefore, in our institution, all milk samples from seropositive mothers of preterm infants at risk are inactivated and raw milk is only rarely available for analysis. Our special interest in the analysis of breast milk cells is based on the fact of compartmentalized viral reactivation during lactation and the hypothetical link between cellular immune control and the phenomenon of decreasing viral shedding during a unimodal course. Therefore, breast milk lymphocyte dynamics was studied using polychromatic flow cytometry during lactation in a cohort of 15 mothers because in blood, lymphocytes are the main actors in the cellular immune response to HCMV (32, 33). Therefore, we used an adapted antibody panel of our imaging flow cytometry approach. We found that CD3+ T cells increased significantly in HCMV-seropositive but not -seronegative individuals. This finding is consistent with the data of Moylan et al., (34) who reported higher leukocyte counts when HCMV-DNA was detectable in breast milk (23).Our results further suggest that increases of previously reported total breast milk leukocytes might be caused or mostly driven by HCMV-induced alterations of the abundance of the breast milk T cell pool, similar to the well-known effects of HCMV on the distribution of peripheral blood T cells (35–37). Furthermore, in an earlier study, breast milk was reported to contain higher frequencies of activated (HLA-DR+) CD8+ effector memory T cells compared to blood, but that study did not provide information on HCMV serostatus (38). Along these lines, we found that frequencies of activated T cells might be even higher in HCMV-seropositives than in -seronegatives at sampling time T2, consistent with the hypothesized association of breast milk T cells with local HCMV reactivation in the mammary gland. Thus, future CD4 and CD8 T cell subset analyses are warranted to determine presumed associations between those and local HCMV reactivation in the mammary gland.

Interestingly, in our study cohort of HCMV-IgG-serotyped breastfeeding mothers, NK cells did not show any dynamic changes during lactation over the observation period, and neither did they differ depending on HCMV-serostatus. However, B cells and NKT-like cells increased significantly from T1 to T2 in seropositive mothers. Similar to our findings for NK cells, Trend et al., (39) studied breast milk cells without regard to HCMV-serostatus and also reported no significant differences in NK cell frequencies in colostrum relative to transient and mature milk. In contrast to our findings, B cells did not show any dynamics in their study, which could be explained by the missing link to the maternal HCMV serostatus.

Regarding our longitudinal investigation of two mothers (HCMV+ and HCMV-), an increase of NK cells in the seropositive mother at the time of peak viral load was detectable. The increase of NK cells in step with the viral load of this one HCMV-seropositive longitudinal participant might be a natural response to HCMV infected cells in the mammary gland. In future studies more and closer time ranges should be investigated to detect peak viral load and corresponding breast milk leukocytes of each mother.

There are certain limitations to this preliminary study. One is clearly the small number of subjects that could be recruited. Another is that data were only available at one time point from some mothers. Any investigations with breast milk of preterm infants and also term infants in neonatology departments is accompanied by major logistical problems with respect to the daily changing maternal presence in the ward and availability of sufficient pumped raw breast milk for investigational purposes. Thus, these constraints are an inherent limitation when collecting breast milk from mothers of preterm infants from the NICU for multiple cellular analysis (13). In our study, samples from a wide range of gestational ages was acquired. Trend et al., (39) reported that breast milk cells did not differ greatly between preterm and full-term infants, implying that for breast milk cell analysis, gestational age does not influence the results.

In conclusion, to the best of our knowledge this study is the first attempting to link cellular morphology using cell imaging to defined leukocyte subtype analysis by flow cytometry approaches in HCMV-seronegative-vs-seropositive breastfeeding mothers during HCMV reactivation. Despite logistical challenges, this work should encourage further studies on the dynamics and function of compartmentalized lymphocytes from milk versus blood lymphocytes in the context of local viral reactivation.

## Data Availability Statement

The raw data supporting the conclusions of this article will be made available by the authors, without undue reservation.

## Ethics Statement

The studies involving human participants were reviewed and approved by Clinical Ethics Committee at the University Hospital Tübingen (804/2015BO2). The patients/participants provided their written informed consent to participate in this study.

## Author Contributions

RG, GP, TK, KH, and KW-H: conceptualization of the study. TK and KW-H: investigation and methodology, KL, SP, and KW-H: formal analysis, data curation and visualization, GP, KH, and KW-H: supervision and project administration, KH and KW-H: resources, KL: writing-original draft preparation, KH, GP, KW-H, SP, and TK: writing-review and editing. All authors contributed to the article and approved the submitted version.

## Funding

The research was funded by a medical thesis grant for TK from IZKF Promotionskolleg, University Hospital Tübingen (PK2015-2-27) and a grant for KL from ‘Sonderlinie Medizin: Verbundprojekt Freiburg-Tübingen-Ulm’ by the Ministry of Science and Art of Baden Wuerttemberg; grant number [KL 2528-0-0].

## Conflict of Interest

The authors declare that the research was conducted in the absence of any commercial or financial relationships that could be construed as a potential conflict of interest.

## Publisher’s Note

All claims expressed in this article are solely those of the authors and do not necessarily represent those of their affiliated organizations, or those of the publisher, the editors and the reviewers. Any product that may be evaluated in this article, or claim that may be made by its manufacturer, is not guaranteed or endorsed by the publisher.

## Supplementary Material

Supplementary Figure 1 Gating strategy of breast milk cells in FlowJo.

Supplementary Figure 2 Breast milk lymphocyte frequencies of either HCMV IgG-seropositive or seronegative subcohorts. Mothers with consecutive samples are color coded (blue: mother 7, red: mother 14 and green: mother 15). Statistical analysis was performed by Mann-Whitney U-test.
