# Supplementary figures and images for: Immunomonitoring of Human Breast Milk Cells During HCMV-Reactivation
Source: Front Immunol. 2021 Sep 9;12:723010. doi: 10.3389/fimmu.2021.723010 (PMC8462275; doi:10.3389/fimmu.2021.723010)

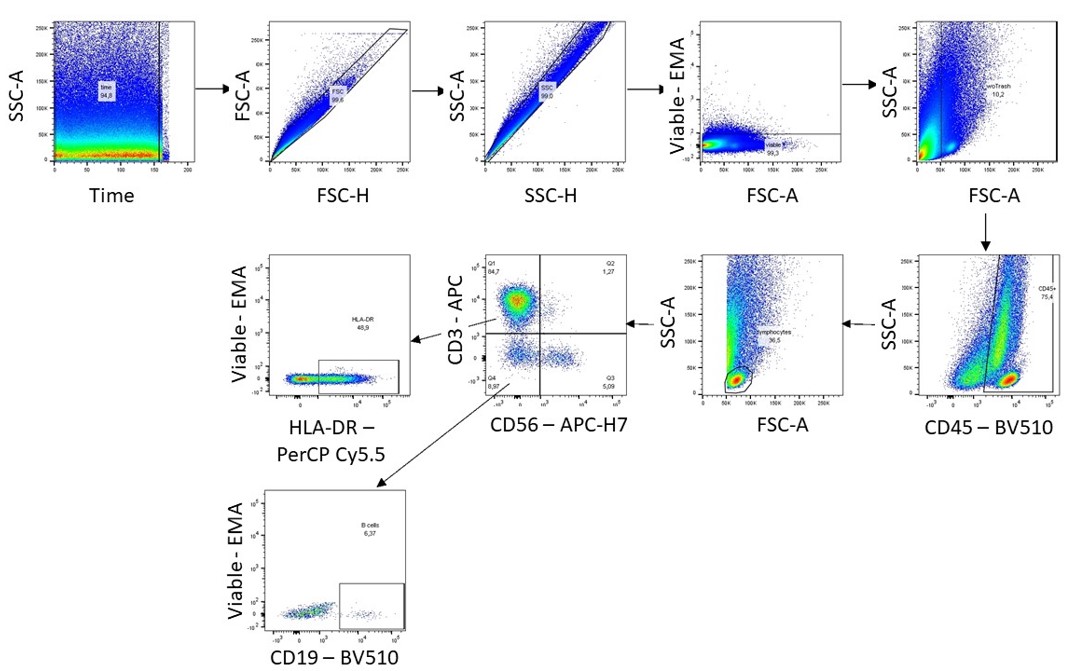

Supplement: Supplementary Figure 1 — Gating strategy of breast milk cells in FlowJo. [file Image_1.jpeg]

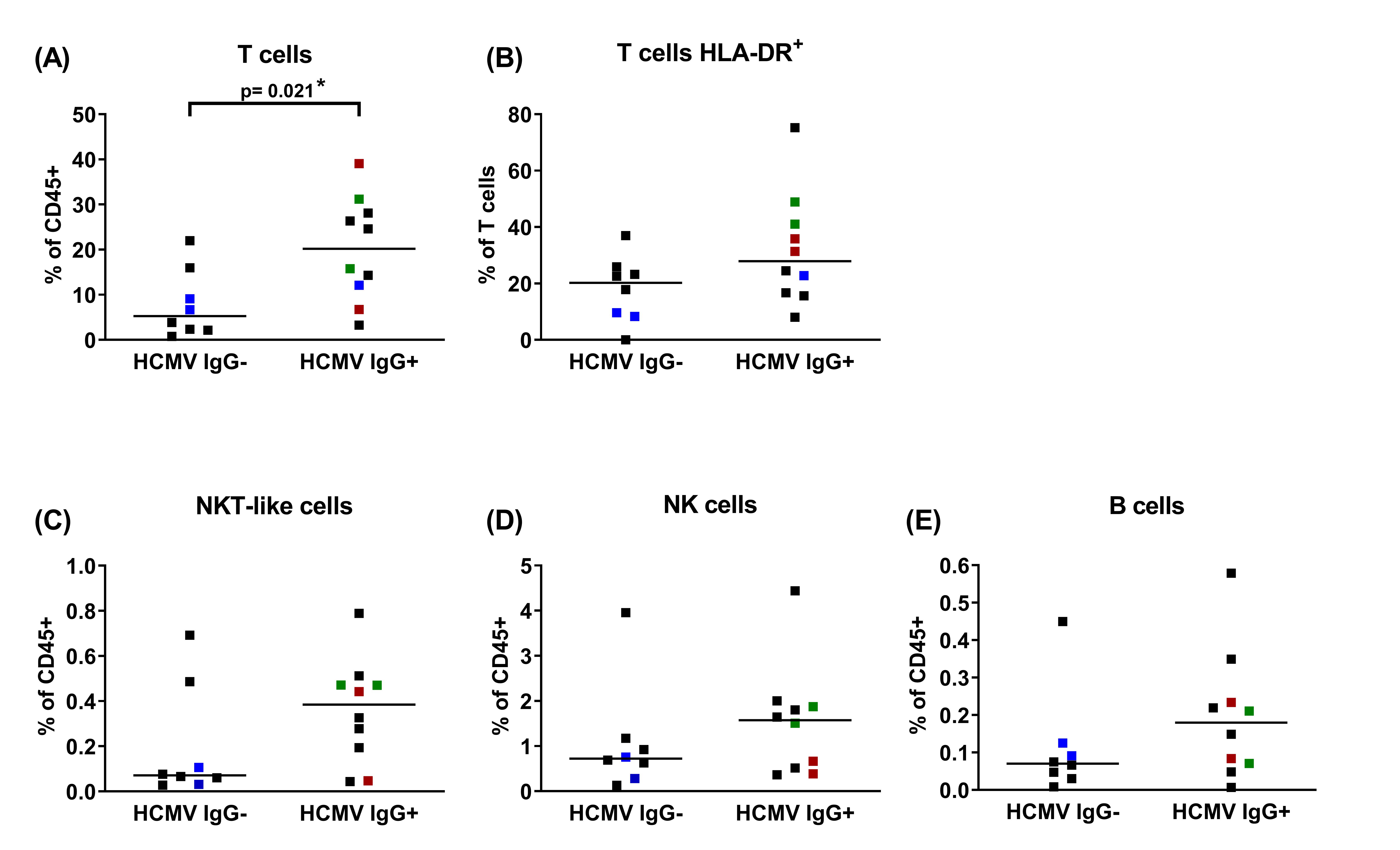

Supplement: Supplementary Figure 2 — Breast milk lymphocyte frequencies of either HCMV IgG-seropositive or seronegative subcohorts. Mothers with consecutive samples are color coded (blue: mother 7, red: mother 14 and green: mother 15). Statistical analysis was performed by Mann-Whitney U-test. [file Image_2.jpeg]
