# Supplementary material for: Immunomonitoring of Human Breast Milk Cells During HCMV-Reactivation
Source: Front Immunol. 2021 Sep 9;12:723010. doi: 10.3389/fimmu.2021.723010 (PMC8462275; doi:10.3389/fimmu.2021.723010)
Supplement: Supplementary file 3 [file Table_1.docx]

**Supplementary Table 1**: Synopsis of breast milk lymphocyte frequency data of all 15 mothers. IQR, inter quartile range. Only subpopulations with parental populations > 200 cells were used for analysis. Mothers 1-7 are CMV IgG-, Mothers 8-15 are CMV IgG+. Bold numbers illustrate T1 and T2, respectively.

| Mother | BM sampling  [days pp] | CD3+  T cells [%] | CD3+ T cells IQR | CD3+ HLA-DR+ T cells [%] | CD3+, HLA-DR IQR | CD56+, CD3+ NKT-like cells [%] | NKT-like cells IQR | CD56+ NK cells [%] | NK cells IQR | CD19+ B cells [%] | B cells IQR | Cohorts |
| --- | --- | --- | --- | --- | --- | --- | --- | --- | --- | --- | --- | --- |
| 1 | 11 | 15.96 | 5.26 | 23.24 | 6.99 | 0.69 | 0.20 | 1.17 | 0.25 | 0.07 | 0.04 | T1 |
| 2 | 12 | 2.35 |  | 22.60 |  | 0.06 |  | 0.69 |  | 0.03 |  | T1 |
| 3 | 16 | 3.87 |  | 17.80 |  | 0.03 |  | 0.92 |  | 0.05 |  | T1 |
| 4 | 44 | 21.98 | 9.21 | 36.97 | 22.42 | 0.49 | 0.12 | 3.96 | 1.13 | 0.45 | 0.13 | T2 |
| 5 | 57 | 0.79 |  | 0 (<LOD) |  | 0.08 |  | 0.63 |  | 0.01 |  | T2 |
| 6 | 78 | 2.15 |  | 25.93 |  | 0.07 |  | 0.13 |  | 0.07 |  | T2 |
| 7 | **12**, 41, 47, **54**, 67 | **6.67,** 25.72, 2.63, **9.09,** 1.79 | **5.26, 9.21** | **9.66,** 22.19, 6.03**, 8.28,** 7.87 | **6.99, 22.42** | **0.11**, 0.10, 0, **0.03**, 0 | **0.20, 0.12** | **0.75,** 0.57, 0.25, **0.28,** 0.18 | **0.25, 1.13** | **0.09,** 0.14, 0.02, **0.13,** 0.04 | **0.04, 0.13** | **T1,T2,** longitudinal |
| 8 | 6 | 28.10 | 6.63 | 8.00 | 14.36 | 0.28 | 0.16 | 1.80 | 1.21 | 0.15 | 0.15 | T1 |
| 9 | 12 | 12.09 |  | 22.78 |  | 0.33 |  | 1.64 |  | 0.22 |  | T1 |
| 10 | 17 | 14.32 |  | 15.60 |  | 0.19 |  | 0.36 |  | 0.01 |  | T1 |
| 11 | 20 | 3.27 |  | 24.48 |  | 0.04 |  | 0.52 |  | 0.05 |  | T1 |
| 12 | 39 | 26.34 | 7.07 | 75.25 | 23.73 | 0.79 | 0.12 | 4.44 | 1.05 | 0.58 | 0.18 | T2 |
| 13 | **108**,  (57, 122, 131) | 24.59 |  | 16.70 |  | 0.51 |  | 2.00 |  | 0.35 |  | T2 |
| 14 | **13**, 22, **42,** 68 | **6.73,** 20.51, **39.07,** 18.41 | **6.63, 7.07** | **31.35**, 34.18, **35.84**, 23.54 | **14.36, 23.73** | **0.05,** 0.15, **0.44,** 0.10 | **0.16, 0.12** | 0.39, 0.32, 0.67, 0.43 | **1.21, 1.05** | **0.08,** 0.23, **0.23,** 0.16 | **0.15, 0.18** | **T1,T2,** longitudinal |
| 15 | **20**, 48, 55, **62** | **15.78,** 18.55, 6.05, **31.18** | **6.63, 7.07** | **41.00,** 48.59, 45.46, **48.91** | **14.36, 23.73** | **0.47**, 0.51, 0.08, **0.47** | **0.16, 0.12** | **1.51,** 2.96, 0.72, **1.87** | **1.21, 1.05** | **0.07,** 0.23, 0.03, **0.21** | **0.15, 0.18** | **T1,T2,** longitudinal |
